# Effects of short-term, high-dose cocoa-derived flavanol supplementation on gut microbiota composition: secondary findings from a randomized, double-blind, placebo-controlled crossover study

**DOI:** 10.1017/jns.2024.17

**Published:** 2024-07-30

**Authors:** Cassandra Suther, Billie Alba, Beau R. Yurkevicius, Patrick N. Radcliffe, Heather S. Fagnant, John Castellani, J. Philip Karl

**Affiliations:** 1 Military Nutrition Division, United States Army Research Institute of Environmental Medicine, Natick, MA, USA; 2 Thermal and Mountain Medicine Division, United States Army Research Institute of Environmental Medicine, Natick, MA, USA; 3 Oak Ridge Institute for Science and Education, Oak Ridge, TN, USA

**Keywords:** Chocolate, High-throughput Sequencing, Microbiome, Polyphenol

## Abstract

Cocoa-derived flavanols (CDF) may act as prebiotics. However, evidence is inconsistent, and the duration and dose of CDF intake needed to elicit any prebiotic effect are undefined. This randomized, double-blind, crossover study determined the effects of short-term, high-dose dietary supplementation with CDF versus matched placebo on gut microbiota composition in 8 healthy adults. A single faecal sample was collected 8 d after supplementation with 900 mg/d CDF or placebo. Gut microbiota composition and *Bifidobacterium* spp. and *Lactobacillus* spp. abundance were measured as secondary outcomes by 16S ribosomal ribonucleic acid (rRNA) amplicon sequencing and quantitative polymerase chain reaction, respectively. No between-treatment differences in the relative or absolute abundance of *Bifidobacterium* spp. (Cohen’s *d* = 0.89, *P* = 0.22) or *Lactobacillus* spp. (Cohen’s *d* = 0.42, *P* = 0.65) were detected. Shannon diversity (Cohen’s *d* = 0.38, *P* = 0.04) and overall community richness (Cohen’s *d* = 0.34, *P* = 0.06) were lower following CDF supplementation versus placebo, but no between-treatment differences in β-diversity or taxa relative abundances were observed. Findings are not consistent with a clear prebiotic effect of this short-term, high-dose CDF supplementation strategy relative to placebo.

## Introduction

The gut microbiota is increasingly recognized as an important mediator of human health.^(1)^ One approach to favourably modulating the gut microbiota is by consuming prebiotics, defined as substrates that are selectively utilized by host microbes conferring a health benefit.^(2)^ Although some controversy surrounds what substrates can be classified as prebiotics, several have promising but incomplete supporting evidence.^(2)^ These “candidate prebiotics” include polyphenols.^(2)^ Polyphenols are secondary plant metabolites that are poorly absorbed in the small intestine, but undergo enzymatic/bacterial metabolism in the colon.^(3)^ Certain polyphenols may favourably modulate the gut microbiota by promoting the growth of beneficial bacteria including *Bifidobacterium* and *Lactobacillus* spp.,^(4,5)^ while reducing abundance of potentially harmful microbes such as *Clostridium perfringens*,^(6)^ which is consistent with a prebiotic effect.^(2)^ However, over eight-thousand polyphenolic compounds have been identified in plant species, and the effects of these compounds on the gut microbiota vary.^(3)^

Flavonoids are the most studied group of polyphenols. The subclass flavanols, specifically the monomeric flavanols catechin, epicatechin, epigallocatechin, gallocatechin, and their gallate derivatives, are found in abundance in tea and cocoa.^(3)^ Initial studies conducted in in vitro models, animals, and healthy humans reported that cocoa-derived flavanols (CDF) favourably impacted the gut microbiota by selectively increasing absolute abundance of beneficial genera *Lactobacillus* and *Bifidobacterium*.^(4,5,7,8)^ In contrast, results of more recent human trials utilizing modern high-throughput sequencing that relies on compositional analysis (i.e. relative abundance), rather than absolute quantification, have failed to observe increases in the relative abundance of *Lactobacillus* or *Bifidobacterium.* Instead, they reported changes in other taxa that are not consistently recognized as beneficial.^(8,9)^ Reasons for the inconsistency across studies are unclear but could be attributed to differences in the microbiota measurement methods, study population, or supplementation strategy, including CDF source, dose, and duration of supplementation. For example, several studies used whole chocolate or cocoa supplementation which contain other compounds known to modulate the gut microbiome, like fibre, caffeine, and theobromine,^(1,10)^ rather than isolated CDF. Additionally, recent studies have not measured absolute abundance of gut microbes, including *Lactobacillus* or *Bifidobacterium*, as is necessary to support prebiotic effects.^(2)^ Thus, more research is needed to substantiate any prebiotic effect of CDF.

Notably, previous CDF or cocoa supplementation trials relied on long duration pre and post supplementation measurements, ranging in duration from 3 to 10 weeks, with no assessment of the minimal time to achieve an effect.^(5,8,9)^ Other candidate prebiotics are known to alter the gut microbiota within a week of starting consumption.^(11)^ Determining whether short-duration (e.g. ∼1 week) supplementation strategies have prebiotic effects may impact cost:benefit decisions within certain populations. These include military personnel, in whom constrained diets may be consumed for only short periods of time.^(12)^ Prebiotics may also be most cost effective before exposure to environments wherein prebiotic health benefits may be maximized.^(13)^ Therefore, this study aimed to determine the effects of short-term dietary supplementation with CDF at a higher dose (900 mg/d) than used in previous studies on gut microbiota composition and abundance of *Lactobacillus* or *Bifidobacterium* in generally healthy adults. We hypothesized this regimen would not result in substantive differences in gut microbiota community composition but would lead to significantly higher absolute and relative abundances of *Lactobacillus* and *Bifidobacterium* following the CDF treatment compared to following the placebo treatment.

## Methods

### Participants

Participants 17–49 years were recruited from the Natick, MA area between January 2020 and October 2021. Study exclusion criteria included antibiotic use within three months; gastrointestinal disease; <4 bowel movements weekly; regular use of medications impacting gastrointestinal function; colonoscopy within three months; inability to avoid non-steroidal anti-inflammatory medications; vegetarian diet; inability or unwillingness to not consume fermented products for 2 weeks prior and through study participation; and actively trying to lose or gain weight. Participants were instructed to discontinue use of any probiotic, prebiotic, or other dietary supplements (excepting multi-vitamins) and refrain from consuming cocoa-based products and flavanol-rich foods for 2 week prior to and throughout the study. If participants routinely ingested tea or coffee, instruction was given to maintain the usual intake throughout the study.

This study was conducted according to the guidelines laid down in the Declaration of Helsinki, and all procedures involving human subjects/patients were approved by the US Army Medical Research and Development Command Institutional Review Board (approval number M-10762). Written informed consent was obtained from all subjects/patients. The trial was registered on www.clinicaltrials.gov as NCT04359082.

### Study design

The data reported herein were included as secondary outcomes in a trial designed to determine the effects of CDF on cold-induced vasodilation and thermoregulatory responses.^(14)^ The study followed a randomized, double-blind crossover design consisting of two eight-day phases with a minimum two-week wash out. Upon enrollment, participants were randomized using computer-generated randomization. Medical staff otherwise unaffiliated with the study then assigned each participant to receive daily supplementation of CDF (225 mg/pill, CocoaVia, Mars, Inc.) and then placebo (100 mg dextrose/pill, Compounded Solutions in Pharmacy, LLC, Monroe, CT) or vice versa, administered in capsule form. The capsules were matched on both caffeine (10 mg/pill) and theobromine (30 mg/pill) content. Participants consumed four pills every morning approximately one hour after breakfast every day throughout each eight-day study phase. When feasible, pills were consumed under the direct supervision of medical staff not involved in data collection. When visual confirmation was not feasible, verbal confirmation of adherence was obtained daily. The dose and duration of CDF supplementation were based on primary study outcomes relating to the effects of CDF on manual dexterity during cold exposure. Study staff and participants were all blinded.

Participants maintained a 4-day food record during days 4–7 of each phase and were asked to maintain a similar diet during both study phases. Research dietitians provided instructions to participants, reviewed all food records to ensure compliance with study food restrictions, and entered data into Food Processor (ESHA Research, Salem, OR) for analysis of nutrient intakes.

### Faecal sample collection and sequencing

A single faecal sample was collected during a 48-hr period between days 6 and 8 of each experimental period. All samples were collected into plastic collection containers, transported at room temperature, and aliquoted within 12 hr of collection. Aliquots were immediately frozen and stored at −80°C until analysis.

#### 16S rRNA amplicon sequencing

Samples were processed and analysed with the ZymoBIOMICS Targeted Sequencing Service (Zymo Research, Irvine, CA). Deoxyribonucleic acid (DNA) was extracted using the ZymoBIOMICS®-96 MagBead DNA Kit. Both positive and negative controls were included in sequence runs. Primers designed to amplify the V3–V4 region of the 16S rRNA gene were used for PCR amplification, and all samples were sequenced in triplicate on the Illumina MiSeq platform (Illumina, Inc., San Diego, CA). Deconvoluted sequences were processed using the DADA2 pipeline with default parameters to obtain amplicon sequence variants (ASVs).^(15)^ Potential sequencing errors and chimeric sequences were removed. Taxonomy assignment was performed using Uclust from Qiime v.1.9.1 using the Zymo Research Database.^(16)^

The median read count for each sample was 33,510 reads (range: 24,722–38,981 reads/sample). Possible sequencing errors were filtered to remove ASV with fewer <2 counts in ≥10% of samples. Remaining reads were classified into 405 unique ASVs assigned to 89 unique genera. For diversity analyses, samples were rarified to 20,000 reads/sample. Within-sample diversity was calculated in R 4.2.1 by Shannon, Simpson and observed ASVs. Absolute total bacteria abundance was determined using a standard curve technique as part of the ZymoBIOMICS Targeted Sequencing Service. Briefly, a standard curve was generated via quantitative polymerase chain reaction (qPCR) from plasmid DNA containing the 16S gene. The equation generated by the standard curve was used to transform Cq values to a number of gene copies/sample. Genome copies/ul were calculated by dividing the gene copy number by the assumed number of gene copies per genome, which is four. Lastly, the amount of DNA/ul of the sample was calculated using the assumed genome size of 4.64 × 10^6^ (*Escherichia coli*).

Quantitative polymerase chain reaction DNA from faecal samples and isolated bacterial cultures were extracted using the DNeasy PowerSoil Pro Kit (QIAGEN, Inc., Germantown, MD). DNA concentrations were then quantified using a Nanodrop (ThermoFisher Scientific, Waltham, MA). To calculate absolute abundance from qPCR, standard curves using serial dilutions were constructed using DNA from isolated cultures of *Bifidobacterium longum* subsp. *longum* (ATCC-55813) and *Lactobacillus acidophilus* (ATCC-4356). DNA was further amplified using 3 μl of the template with previously published primers^(17)^ and Luna Universal qPCR Kits (New England Biolabs, Ipswich, MA). Amplification was performed for 45 cycles at 95°C for 15s and 60°C for 60s, followed by a melt curve from 60°C to 95°C, using a QuantStudio 6 Flex Real-Time PCR System (ThermoFisher Scientific, Waltham, MA). Genome size for each bacterium was used to calculate qPCR copy number (http://cels.uri.edu/gsc/cndna.html).

### Statistical analysis

Sample size calculations for primary study outcomes determined that 10 participants would be necessary for detecting meaningful and expected mean ± standard deviation (SD) between-treatment differences in skin temperature at the finger (2°C ± 2.3°C)^(18)^ and finger blood flow response (25 units ± 10 units)^(19)^ to cold exposure at α = 0.05 and power = 0.80.^(14)^ Sample size calculations for the secondary outcomes presented here were based on data reported by Tzounis *et al.* ^(5)^ wherein cocoa flavanol supplementation increased abundance of *Bifidobacterium* spp. and *Lactobacillus/Enterococcus* spp. by Cohen’s *d* effect sizes >3. Based on those data, four participants would be needed to detect similar effect sizes at α = 0.05 and power = 0.80.^(14)^ The eight participants included in the present analysis were sufficient to detect a minimum effect size of 1.2 at α = 0.05 and power = 0.80.

Between-treatment differences in α-diversity, absolute abundance, and qPCR copy number were determined using a linear mixed model with a supplement, treatment sequence, their interaction, age, and body mass index (BMI) included as fixed factors, and subject as a random effect, using R packages afex v 1.3.0. Normal distribution and homoscedasticity of residuals were verified for all models. β-diversity was measured using Bray–Curtis dissimilarity, and weighted/unweighted UniFrac distances and were analysed by nested PERMANOVA with the R package microbiome association with linear models (MaAsLin2)^(21)^ (total sum scaling normalization, minimum abundance set to 0.0001, and prevalence set to 0.2) with a supplement, treatment sequence, study phase age, and BMI included as fixed factors. The subject was included as a random effect in the MaAsLin2 model. The Benjamini–Hochberg procedure was used to adjust *P*-values. Data are presented as mean ± SD or median (interquartile range) unless otherwise noted. Statistical significance was defined as *P* ≤ 0.05 and *Q* ≤ 0.20.

## Results and discussion

Previous studies reporting the effects of CDF and cocoa supplementation on the human gut microbiota are inconsistent, with some, but not all, reporting potential prebiotic effects.^(4,5,7,8)^ This randomized, double-blind, placebo-controlled crossover study aimed to extend that evidence by determining the effects of short-term (8 d) and high-dose (900 mg/d) dietary supplementation with CDF versus placebo on the gut microbiota of healthy adults. Eleven volunteers participated in the study, but two were withdrawn from the study and faecal samples could not be collected from a third (Supplemental Fig. 1). Thus, the present analysis includes the seven male and one female volunteer who provided faecal samples following both study phases. Dietary energy and macronutrient intakes were similar between phases (Table 1), and adherence to the intervention was 100% during both phases.

**Table 1.** Baseline demographics and dietary intake

	Placebo	Cocoa flavanol	*P*-value^a^
Male/Female	7/1		
Age (year)	25 ± 5		
BMI (kg/m^2^)	25 ± 4		
Energy (kcal/d)	2481 ± 723	2309 ± 685	0.48
Fat (g/d)	105 ± 46	102 ± 36	0.39
Protein (g/d)	118 ± 44	105 ± 32	0.18
Carbohydrate (g/d)	262 ± 79	239 ± 69	0.84
Fibre (g/d)	20 ± 8	17 ± 6	0.16

a Paired *t*-test (n = 8).

**Fig. 1.**
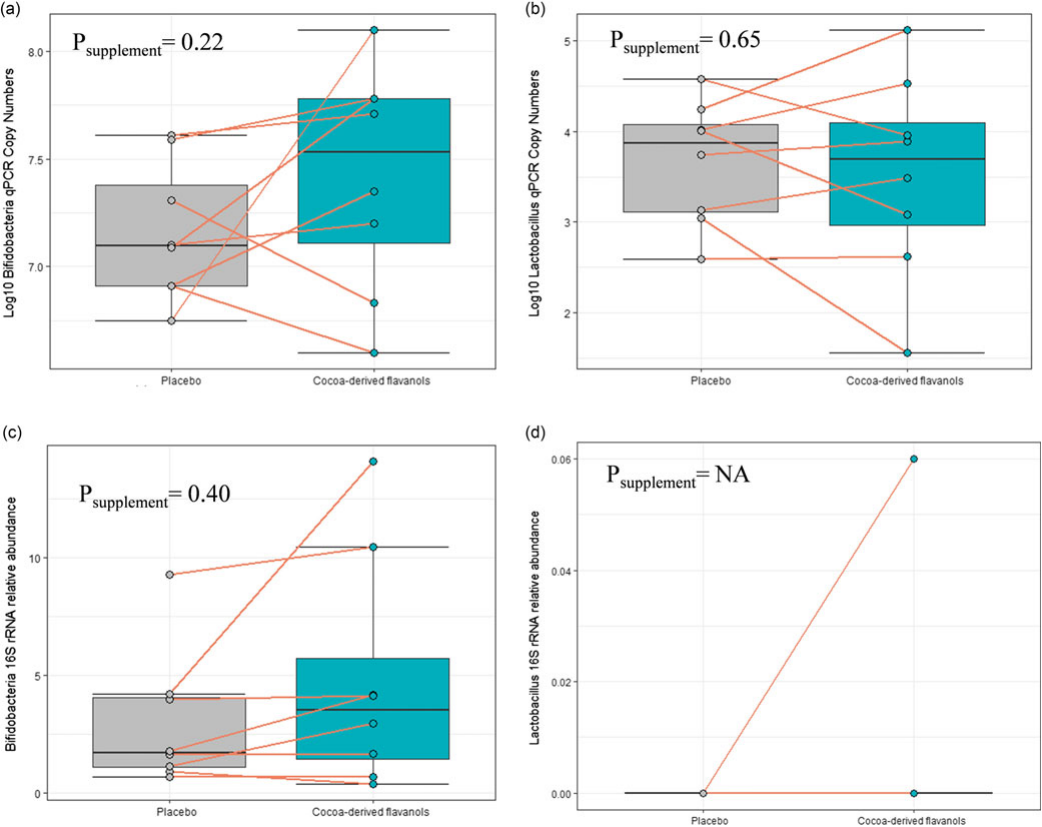
Comparison between log10 qPCR copy numbers (a–b, absolute abundance) and 16S rRNA sequencing (c–d, relative abundance) for *Bifidobacteria* and *Lactobacillus* spp. following eight days of cocoa-derived flavanol supplementation. Bar plots show individual data after placebo and cocoa-derived flavanol supplementation. Individual data are shown. Box plots display median, interquartile range, and range. *Lactobacillus* was only detected in one volunteer via 16S rRNA sequencing.

Faecal samples were analysed using both targeted quantitative and compositional approaches to better substantiate selective growth of the health-promoting genera *Lactobacillus* and *Bifidobacterium.* However, neither higher absolute nor relative abundance of *Lactobacillus* or *Bifidobacterium* were observed following CDF supplementation relative to placebo (Fig. 1, Table 2). Results contrast with previous studies that have reported increases in the absolute abundance of both genera following supplementation with 494 mg cocoa flavanols for 4 weeks in humans and ∼410 mg/d for 4 weeks in pigs.^(4,5)^ Of note, in the present study, *Bifidobacteria* spp. relative and absolute abundance were higher following CDF supplementation relative to placebo in 6/8 volunteers and the mean between-treatment difference in absolute abundance was 0.26 log_10_ copy numbers (95% confidence inrerval (CI): 0.25, 0.78); Fig. 1c). The latter result is similar to that reported by Tzounis *et al.*, wherein CDF supplementation increased the growth of *Bifidobacterium* by ∼0.2 log_10_ units more than the placebo group when compared to baseline samples.^(5)^ Comparisons between that study and this study are complicated by Tzounis *et al.*’s use of change from baseline in the analysis whereas this study relied solely on post-treatment values. Nonetheless, the similarities in effect sizes suggest that an effect of CDF on *Bifidobacterium* spp. in this study should not be ruled out and may have been detected with a larger sample size, higher dosage, or longer duration supplementation period.

**Table 2.** Differential abundance analysis for select genera^a^

Genus	Group	Relative abundance (%)^b^	*P*-value, *Q*-value	Cohen’s *d*
*Lactobacillus* ^c^ ^(5)^	CDF	0.00 (0.00)	DeSeq2- N/A MaAsLin2- N/A	–
	Placebo	0.00 (0.00)		
*Bifidobacterium* ^(5)^	CDF	3.55 (4.30)	DeSeq2- 0.11, 1.0 MaAsLin2- 0.24, 0.82	0.46
	Placebo	1.72 (2.95)		
*Blautia* ^(8,9)^	CDF	12.64 (4.08)	DeSeq2- 0.89, 1.0 MaAsLin2- 0.56, 0.87	0.28
	Placebo	13.38 (4.72)		
*Lachnospira* ^(8)^	CDF	0.33 (2.64)	DeSeq2- 0.28, 1.0 MaAsLin2- 0.75, 0.94	0.60
	Placebo	0.61 (2.85)		
*Clostridium* ^(5)^	CDF	0.02 (0.07)	DeSeq2- 0.68, 1.0 MaAsLin2- 0.73, 0.94	0.39
	Placebo	0.00 (0.08)		
*Faecalibacterium* ^(9)^	CDF	12.41 (2.35)	DeSeq2- 0.17, 1.0 MaAsLin2- 0.22, 0.82	0.55
	Placebo	10.31 (4.27)		

n = 8. CDF, cocoa-derived flavanols.

a Genera selected are those that have been reported to be altered by cocoa-derived flavanol supplementation or cocoa products in clinical trials. See Supplemental Tables 1 and 2 for full results.

b Reported as median (interquartile range).

c Detected in only one volunteer via 16S rRNA amplicon sequencing throughout the study.

Most participants had no *Lactobacillus* detected by 16S rRNA amplicon sequencing (Fig. 1d), which may be due to the inherent bias in the V3–V4 primers used.^(22)^ All participants did have *Lactobacillus* counts detected via qPCR. That discrepancy highlights one limitation of relying solely on 16s rRNA sequencing when assessing candidate prebiotics. The observed differences between relative and absolute abundance of *Lactobacillus* may also be due to the qPCR primers amplifying low abundance, food-related bacteria within the genera *Pediococcus*, *Leuconostoc*, and *Weissella*.^(17)^ Although none of those taxa were identified with via amplicon sequencing. Thus, low baseline abundance may be one reason why the effects of CDF on *Lactobacillus* were not detected. A 700% increase in faecal *Lactobacillus* (current taxonomy: *Lacticaseibacills*) *casei* abundance was reported by Jang *et al.* following CDF supplementation in a pig model, but no difference in the abundance of other *Lactobacillus* species were detected.^(4)^ It is possible that *L. casei* was not present within our cohort or other *Lactobacillus* species were detected, leaving *L. casei*-specific growth undetectable. Differences in the primer or nucleic targets used may also explain differences in the present results relative to previous studies. Specifically, the oligonucleotides used by Tzounis *et al.* in their study of CDF supplementation simultaneously detected both *Lactobacillus* and *Enterococcus* spp.^(5)^ The primers used in this study are not reported to amplify *Enterococcus* spp. Alternately, the shorter duration of CDF supplementation in this study compared to the 4 weeks duration used by Jang *et al.* and Tzounis *et al.* may not have been long enough to promote growth of *Lactobacillus* spp. Whether CDF do indeed promote the growth of multiple *Lactobacillus* spp. or only a select subset requires further research. Future research should also consider the revised taxonomic groupings that have reclassified *Lactobacillus* into 25 separate genera.^(17)^

**Fig. 2.**
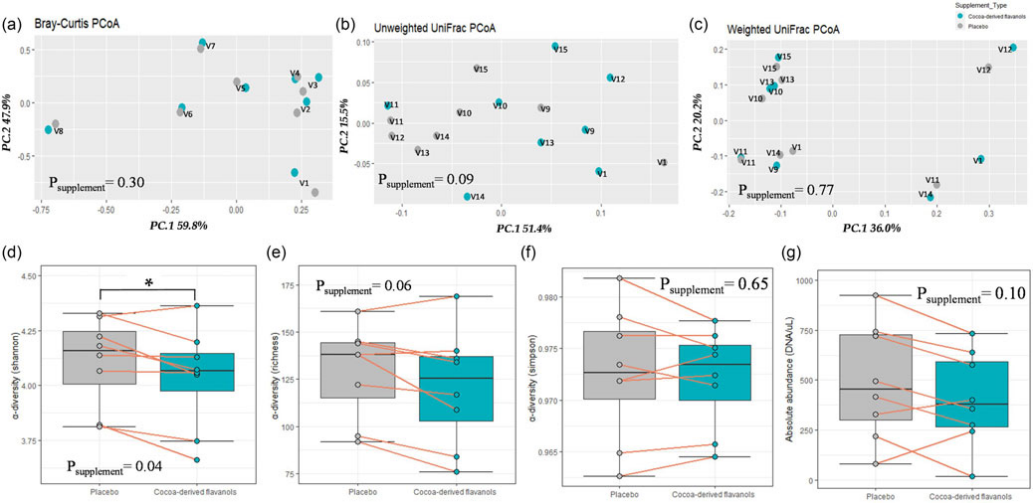
Ecology diversity, richness, and absolute abundance following eight days of cocoa-derived flavanol supplementation. Diversity measures (a) Bray–Curtis, (b) unweighted UniFrac, (c) weighted UniFrac, (d) Shannon, (e) Simpson, (f) richness, and (g) absolute abundance. Individual data are shown. Box plots display median, interquartile range, and range. Data analysed by nested permutational analysis of variance (PERMANOVA) (a–c) or linear mixed model (d–g) with supplementation, sequence, their interaction, age and BMI as fixed effects, and subject as a random intercept/restricted permutation.

When diversity was examined, the presence of rare taxa in the community did appear to be lower following CDF supplementation relative to placebo. Specifically, Shannon diversity (mean difference (95% CI) = –0.08 (0.003, 0.15), Cohen’s *d* = 0.38; *P*_supplementation_ = 0.04), and overall community richness (–9 (–19, 2), Cohen’s *d* = 0.34; *P*_supplementation_ = 0.06) were lower. Total DNA content (mean difference (95% CI) = –86 (21, 192) and Cohen’s *d* = 0.35; *P*_supplementation_ = 0.10) demonstrated a tendency to be lower following CDF supplementation compared to placebo (Fig. 2d–g). Shannon’s index is more sensitive to the number of rare species in a community than the Simpson index, which is influenced more by dominant taxa, and which did not differ between CDF supplementation and placebo (Fig. 2f). Additionally, although no significant between-treatment differences were observed for any of the β-diversity metrics, dissimilarity between samples collected from the same individuals was visually apparent in the unweighted UniFrac analysis (*P* = 0.09) which relies on the presence and absence of taxa rather than their relative abundance which are used by the other metrics (Fig. 2a–c; *P*_supplementation_ ≥ 0.20). Of note, broad-spectrum antibiotics are known to decrease overall diversity, and polyphenols are known antimicrobials that have been added to food products to increase shelf life.^(1,7)^ Specifically, flavonoids have been shown to inhibit several pathogenic bacteria in both gram stain groups, including *Staphylococcus aureus, Vibrio cholerae, Streptococcus mutans, Clostridium perfringens, Clostridium difficile, Streptococcus pyogenes, and Escherichia coli*.^(6)^ Studies in rats have also reported decreases in the genera *Clostridium* and *Staphylococcus* following cocoa-flavanol supplementation.^(23)^

Differences in α-diversity metrics following CDF supplementation relative to placebo contrasted with an absence of any observed differences in genus relative abundances (Table 2). No differences in genus relative abundances between CDF supplementation and placebo were observed in either DeSeq2 or MaAsLin2 models (*Q* > 0.2) (Supplemental Tables 1 and 2). When considered along with the diversity results, these findings suggest that specific rare species were decreased following the CDF supplementation relative to placebo. However, those differences were not detected in differential abundance analyses likely due to limitations of 16S rRNA sequencing or the filtering approach/statistical models used for analysis.

Two previous studies using lower doses of CDF-containing products (132–425 mg/d CDF) for longer durations (3–10 weeks) and 16S rRNA amplicon sequencing reported either no effect or an increase in alpha-diversity and variable effects on the relative abundance of several taxa including *Blautia, Lachnospira,* and *Faecalibaterium* (Table 2).^(8,9)^ Reasons for the inconsistencies across these studies could be due to not having baseline gut microbiota comparisons in the present study, the dose or duration of CDF supplementation, or the type of supplement used. Regarding the latter, previous studies supplemented with 85% dark chocolate^(9)^ or cocoa-powder^(8)^ contain not only CDF but also other microbiota-modulating compounds such as caffeine, theobromine, and fibre. Importantly, the provided placebos did not contain any of those compounds. Thus, whether CDF were solely responsible for observed effects in those studies is unclear.

Strengths of this study included the double-blind crossover design, an attempt to isolate the effects of CDF by matching of theobromine and caffeine content in the placebo and intervention products, and the use of both compositional and quantitative microbiota measures. Several limitations warrant consideration. First, this study did not include a baseline time point. Rather, all comparisons were made between samples collected at the end of the supplementation phases. This precludes comparing changes in gut microbiota composition during each phase or assessing whether changes during the first phase returned to baseline prior to starting the second phase. As such, results may not be directly comparable to previous studies assessing changes from baseline. Second, the small sample size, although adequate to detect previously reported effect sizes,^(5)^ reduced the power to detect effect sizes measured herein as variability was higher than in previous reports. The higher variability was likely due in part to collecting only one sample during each study period, preventing an assessment of change in composition within each period. Third, the predominantly male cohort reduces generalizability. Finally, the supplementation period may not have been sufficiently long enough to elicit measurable changes within the gut microbiota. However, studying this brief supplementation period is warranted as previous studies have reported diet and diet supplement-induced shifts in the gut microbiota within days.^(11)^ Importantly, this intervention duration has practical implications for certain populations such as military personnel.

In summary, this study investigated the effects of dietary supplementation with a high dose (900 mg/d) of CDF for 8 days on gut microbiota composition compared with a placebo. Within that context, a lower diversity of the gut microbiota was observed following CDF supplementation compared to placebo, which suggested a reduction in rare taxa. No between-group differences in the differential abundance of any taxa, including those previously reported to be affected by CDF, dark chocolate, or cocoa powder, were observed. However, an effect of CDF on *Bifidobacterium* could not be ruled out and may have been detected with a larger sample size or longer duration supplementation period. Findings therefore indicate that additional investigations into the prebiotic potential of CDF and the dose and duration of supplementation required to elicit any prebiotic effect are warranted.

## Supporting information

Suther et al. supplementary material Suther et al. supplementary material

## Supplementary material

For supplementary material accompanying this paper visit https://doi.org/10.1017/jns.2024.17. click here to view supplementary material
