# Supplementary material for: Effects of short-term, high-dose cocoa-derived flavanol supplementation on gut microbiota composition: secondary findings from a randomized, double-blind, placebo-controlled crossover study
Source: J Nutr Sci. 2024 Jul 30;13:e22. doi: 10.1017/jns.2024.17 (PMC11704926; doi:10.1017/jns.2024.17)
Supplement: Suther et al. supplementary material [file S204867902400017Xsup001.docx]

| Table S1. MaAsLin2 output between placebo and cocoa-flavanol supplementation. | | | | | | |
| --- | --- | --- | --- | --- | --- | --- |
| Feature | coef | stderr | N | N.not.0 | pval | qval |
| *Ruminococcus* | 0.451715 | 0.156843 | 16 | 10 | 0.028056 | 0.666013944 |
| *Tyzzerella* | 0.969929 | 0.444276 | 16 | 5 | 0.053956 | 0.666013944 |
| *Phascolarctobacterium* | 0.263093 | 0.127575 | 16 | 9 | 0.084791 | 0.666013944 |
| *Odoribacter* | 0.566303 | 0.284411 | 16 | 14 | 0.093567 | 0.666013944 |
| *Gordonibacter* | -1.10061 | 0.619823 | 16 | 5 | 0.10617 | 0.666013944 |
| *Intestinibacter* | 1.481288 | 0.782455 | 16 | 5 | 0.107186 | 0.666013944 |
| *Eggerthella* | 0.894829 | 0.511724 | 16 | 6 | 0.110922 | 0.666013944 |
| *Prevotella* | -0.68028 | 0.365478 | 16 | 6 | 0.112015 | 0.666013944 |
| *Holdemanella* | 0.194942 | 0.112901 | 16 | 4 | 0.134977 | 0.666013944 |
| *Fusicatenibacter* | -0.45457 | 0.263309 | 16 | 16 | 0.135031 | 0.666013944 |
| *Lachnospiraceae* | 0.378908 | 0.224262 | 16 | 16 | 0.142069 | 0.666013944 |
| *Butyricimonas* | 0.64831 | 0.387257 | 16 | 5 | 0.145133 | 0.666013944 |
| *Eubacterium* | 0.445119 | 0.270481 | 16 | 16 | 0.150935 | 0.666013944 |
| *Allisonella* | 0.097892 | 0.063666 | 16 | 4 | 0.175067 | 0.666013944 |
| *Prevotellaceae* | -0.65103 | 0.432493 | 16 | 8 | 0.182956 | 0.666013944 |
| *Dialister* | 0.627505 | 0.471517 | 16 | 9 | 0.212794 | 0.666013944 |
| *Lachnoclostridium.Roseburia* | -0.60849 | 0.443019 | 16 | 13 | 0.218707 | 0.666013944 |
| *Slackia* | 0.140357 | 0.102813 | 16 | 4 | 0.22117 | 0.666013944 |
| *Faecalibacterium* | -0.24464 | 0.179216 | 16 | 16 | 0.221195 | 0.666013944 |
| *Family.XIII* | 0.454945 | 0.338725 | 16 | 11 | 0.227809 | 0.666013944 |
| *Bifidobacterium* | -0.43731 | 0.338497 | 16 | 16 | 0.243911 | 0.666013944 |
| *Dorea* | -0.14026 | 0.109413 | 16 | 16 | 0.247185 | 0.666013944 |
| *Ruminococcaceae* | 0.961522 | 0.753823 | 16 | 16 | 0.249279 | 0.666013944 |
| *Marvinbryantia* | 0.677462 | 0.576526 | 16 | 13 | 0.28448 | 0.666013944 |
| *Desulfovibrio* | -0.05614 | 0.050069 | 16 | 6 | 0.305009 | 0.666013944 |
| *Bacteroides* | -0.07534 | 0.067767 | 16 | 16 | 0.308793 | 0.666013944 |
| *Adlercreutzia.Asaccharobacter* | -0.3992 | 0.364193 | 16 | 8 | 0.315062 | 0.666013944 |
| *Ruminiclostridium* | 0.648093 | 0.592802 | 16 | 16 | 0.316214 | 0.666013944 |
| *Anaerotruncus* | 0.386983 | 0.359403 | 16 | 11 | 0.322974 | 0.666013944 |
| *Erysipelatoclostridium* | 0.461018 | 0.443017 | 16 | 7 | 0.338151 | 0.666013944 |
| *Coriobacteriaceae* | -0.23019 | 0.22125 | 16 | 8 | 0.338242 | 0.666013944 |
| *Faecalitalea* | 0.324775 | 0.324783 | 16 | 4 | 0.355928 | 0.666013944 |
| *Paraprevotella* | 0.476019 | 0.492133 | 16 | 6 | 0.370771 | 0.666013944 |
| *Alistipes* | 0.243078 | 0.253396 | 16 | 14 | 0.37446 | 0.666013944 |
| *Akkermansia* | -0.38717 | 0.404976 | 16 | 6 | 0.375976 | 0.666013944 |
| *Bilophila* | -0.21261 | 0.237979 | 16 | 11 | 0.406039 | 0.692140329 |
| *Parabacteroides* | 0.331689 | 0.377208 | 16 | 15 | 0.413051 | 0.692140329 |
| *Senegalimassilia* | 0.132627 | 0.157189 | 16 | 6 | 0.43116 | 0.693112777 |
| *Enterorhabdus* | -0.14851 | 0.179547 | 16 | 4 | 0.439796 | 0.693112777 |
| *Coprococcus* | 0.20152 | 0.247815 | 16 | 16 | 0.44717 | 0.693112777 |
| *Streptococcus* | -0.35101 | 0.447798 | 16 | 15 | 0.46294 | 0.700056305 |
| *Sutterella* | -0.416 | 0.552515 | 16 | 11 | 0.479988 | 0.708553379 |
| *Lachnoclostridium* | 0.081802 | 0.115844 | 16 | 16 | 0.506583 | 0.717201934 |
| *Veillonella* | 0.319964 | 0.484641 | 16 | 6 | 0.524035 | 0.717201934 |
| *Romboutsia* | -0.27513 | 0.419525 | 16 | 16 | 0.536262 | 0.717201934 |
| *Erysipelotrichaceae* | 0.236973 | 0.377825 | 16 | 14 | 0.553628 | 0.717201934 |
| *Barnesiella* | 0.090967 | 0.147746 | 16 | 8 | 0.560708 | 0.717201934 |
| *Blautia* | 0.086888 | 0.141624 | 16 | 16 | 0.562058 | 0.717201934 |
| *Flavonifractor* | 0.182597 | 0.302552 | 16 | 6 | 0.56826 | 0.717201934 |
| *Blautia.Lachnoclostridium* | 0.106595 | 0.187204 | 16 | 5 | 0.589747 | 0.717201934 |
| *Escherichia.Shigella* | 0.445328 | 0.800024 | 16 | 5 | 0.597904 | 0.717201934 |
| *Parasutterella* | -0.03932 | 0.071367 | 16 | 4 | 0.601524 | 0.717201934 |
| *Subdoligranulum* | 0.145418 | 0.321003 | 16 | 16 | 0.666461 | 0.779634046 |
| *Christensenellaceae* | 0.162152 | 0.435185 | 16 | 13 | 0.72225 | 0.808336304 |
| *Howardella* | -0.06629 | 0.182577 | 16 | 4 | 0.728978 | 0.808336304 |
| *Clostridium* | 0.159754 | 0.441913 | 16 | 7 | 0.73011 | 0.808336304 |
| *Lachnospira* | 0.199812 | 0.599611 | 16 | 14 | 0.750293 | 0.816108399 |
| *Turicibacter* | -0.06554 | 0.218583 | 16 | 10 | 0.774431 | 0.827839791 |
| *Anaerostipes* | -0.04539 | 0.251156 | 16 | 16 | 0.862533 | 0.893651388 |
| *Collinsella* | 0.049243 | 0.277152 | 16 | 14 | 0.864824 | 0.893651388 |
| *Thalassospira* | 0.029729 | 0.253812 | 16 | 4 | 0.910579 | 0.925506882 |
| *Roseburia* | -0.00997 | 0.326609 | 16 | 16 | 0.976635 | 0.976635049 |
| Placebo is used as reference. Total sum scaling normalization, minimum abundance set to 0.0001 and prevalence set to 0.2. Supplement, treatment sequence, study phase age, and BMI included as fixed factors. Subject included as random effect. | | | | | | |

| Table S2. DESeq2 output between placebo and cocoa-flavanol supplementation | | | | | | |
| --- | --- | --- | --- | --- | --- | --- |
|  | baseMean | log2FoldChange | lfcSE | stat | pvalue | padj |
| *Phascolarctobacterium* | 155.87698 | 24.41959 | 1.37275 | 3.59242 | 0.05804 | 1.00000 |
| *Gordonibacter* | 12.23250 | -6.13908 | 2.92469 | 3.28503 | 0.06991 | 1.00000 |
| *Intestinibacter* | 65.23610 | 22.44300 | 2.86694 | 3.24590 | 0.07160 | 1.00000 |
| *Bifidobacterium* | 1322.68590 | -0.84379 | 0.51541 | 2.63385 | 0.10461 | 1.00000 |
| *Eubacterium* | 2158.42091 | 0.47162 | 0.31992 | 2.15089 | 0.14249 | 1.00000 |
| *Faecalibacterium* | 3791.17158 | -0.25869 | 0.18518 | 1.93164 | 0.16458 | 1.00000 |
| *Blautia.Lachnoclostridium* | 40.20264 | 30.00000 | 2.85981 | 1.75904 | 0.18474 | 1.00000 |
| *Ruminiclostridium* | 225.34208 | 0.86373 | 0.67057 | 1.52296 | 0.21717 | 1.00000 |
| *Butyricimonas* | 10.85746 | 29.83533 | 2.95201 | 1.23490 | 0.26646 | 1.00000 |
| *Romboutsia* | 245.62137 | -0.87410 | 0.78094 | 1.22647 | 0.26809 | 1.00000 |
| *Fusicatenibacter* | 1059.23253 | -0.49938 | 0.45398 | 1.20388 | 0.27255 | 1.00000 |
| *Lachnospiraceae* | 1831.70850 | 0.31309 | 0.29200 | 1.14629 | 0.28433 | 1.00000 |
| *Ruminococcaceae* | 2215.79355 | 0.62382 | 0.59120 | 1.04280 | 0.30717 | 1.00000 |
| *Marvinbryantia* | 67.50321 | 1.27471 | 1.27304 | 0.75676 | 0.38434 | 1.00000 |
| *Faecalitalea* | 5.72072 | 11.14593 | 2.98264 | 0.66917 | 0.41334 | 1.00000 |
| *Lachnoclostridium.Roseburia* | 177.78745 | -0.93866 | 1.13812 | 0.63998 | 0.42372 | 1.00000 |
| *Escherichia-Shigella* | 77.78109 | 2.28170 | 2.93662 | 0.61352 | 0.43347 | 1.00000 |
| *Adlercreutzia.Asaccharobacter* | 28.69338 | -1.78972 | 2.16371 | 0.58840 | 0.44304 | 1.00000 |
| *Tyzzerella* | 18.31460 | 2.15507 | 2.86840 | 0.54968 | 0.45845 | 1.00000 |
| *Family XIII* | 23.22465 | 1.37799 | 1.63061 | 0.54195 | 0.46163 | 1.00000 |
| *Paraprevotella* | 93.67199 | 1.85278 | 2.66191 | 0.45678 | 0.49913 | 1.00000 |
| *Coprococcus* | 553.12332 | 0.38969 | 0.55497 | 0.45436 | 0.50027 | 1.00000 |
| *Bilophila* | 24.38800 | -0.98238 | 1.48823 | 0.38610 | 0.53435 | 1.00000 |
| *Lachnospira* | 215.79854 | 0.65980 | 1.07404 | 0.35314 | 0.55234 | 1.00000 |
| *Subdoligranulum* | 951.04180 | 0.29317 | 0.49711 | 0.34213 | 0.55860 | 1.00000 |
| *Streptococcus* | 177.27243 | -0.45825 | 0.85982 | 0.26228 | 0.60856 | 1.00000 |
| *Odoribacter* | 50.48839 | 0.53741 | 1.04420 | 0.25644 | 0.61258 | 1.00000 |
| *Prevotella* | 1196.68943 | -1.37792 | 2.88574 | 0.21846 | 0.64022 | 1.00000 |
| *Christensenellaceae* | 123.32777 | 0.61147 | 1.22204 | 0.21412 | 0.64356 | 1.00000 |
| *Erysipelatoclostridium* | 29.49891 | 1.12583 | 2.45135 | 0.19436 | 0.65931 | 1.00000 |
| *Clostridium* | 178.38434 | 0.96006 | 2.32516 | 0.16896 | 0.68104 | 1.00000 |
| *Sutterella* | 183.81940 | -0.70865 | 1.63852 | 0.16536 | 0.68427 | 1.00000 |
| *Akkermansia* | 205.48841 | -1.30130 | 2.88613 | 0.16393 | 0.68557 | 1.00000 |
| *Ruminococcus* | 688.31603 | 0.84006 | 2.10111 | 0.16175 | 0.68755 | 1.00000 |
| *Bacteroides* | 2968.61821 | -0.15234 | 0.42940 | 0.12423 | 0.72450 | 1.00000 |
| *Alistipes* | 407.36727 | 0.33326 | 0.98102 | 0.10686 | 0.74375 | 1.00000 |
| *Senegalimassilia* | 42.45078 | 0.89632 | 2.76517 | 0.10648 | 0.74419 | 1.00000 |
| *Dorea* | 830.03096 | -0.12496 | 0.38609 | 0.10359 | 0.74757 | 1.00000 |
| *Parabacteroides* | 332.59391 | 0.20751 | 0.75007 | 0.07533 | 0.78372 | 1.00000 |
| *Prevotellaceae* | 752.57908 | -0.70232 | 2.88597 | 0.05741 | 0.81064 | 1.00000 |
| *Collinsella* | 990.90057 | 0.22672 | 0.99842 | 0.05149 | 0.82049 | 1.00000 |
| *Blautia* | 4313.71283 | 0.02716 | 0.17925 | 0.02271 | 0.88020 | 1.00000 |
| *Anaerostipes* | 440.24392 | -0.06349 | 0.48005 | 0.01720 | 0.89566 | 1.00000 |
| *Dialister* | 341.67667 | -0.10339 | 1.41787 | 0.00960 | 0.92194 | 1.00000 |
| *Erysipelotrichaceae* | 407.42781 | -0.11209 | 1.18082 | 0.00832 | 0.92733 | 1.00000 |
| *Howardella* | 7.67701 | 0.25401 | 2.99443 | 0.00752 | 0.93092 | 1.00000 |
| *Lachnoclostridium* | 924.79904 | 0.03923 | 0.48445 | 0.00648 | 0.93585 | 1.00000 |
| *Roseburia* | 688.51793 | 0.03147 | 0.51391 | 0.00373 | 0.95128 | 1.00000 |
| *Turicibacter* | 95.67444 | 0.08480 | 1.80151 | 0.00223 | 0.96229 | 1.00000 |
| *Allisonella* | 7.58817 | 0.15092 | 2.98707 | 0.00160 | 0.96804 | 1.00000 |
| *Barnesiella* | 80.55008 | -0.05763 | 2.42241 | 0.00056 | 0.98107 | 1.00000 |
| *Anaerotruncus* | 65.81868 | 0.03692 | 1.71548 | 0.00046 | 0.98296 | 1.00000 |
| *Thalassospira* | 45.02274 | -0.26557 | 2.97515 | 0.00000 | 0.99978 | 1.00000 |
| *Holdemanella* | 173.46777 | 0.28340 | 2.63031 | 0.00000 | 0.99997 | 1.00000 |
| *Coriobacteriaceae* | 39.32390 | -0.00248 | 2.06839 | -0.00007 | 1.00000 | 1.00000 |
| *Parasutterella* | 40.13601 | -0.03472 | 2.91660 | -0.00001 | 1.00000 | 1.00000 |
| *Veillonella* | 30.10648 | -1.59461 | 2.61627 | -0.08471 | 1.00000 | 1.00000 |
| *Flavonifractor* | 14.07246 | -13.86326 | 2.54041 | -0.20593 | 1.00000 | 1.00000 |
| *Eggerthella* | 15.47814 | 1.57007 | 2.36409 | -1.12452 | 1.00000 | 1.00000 |
| *Desulfovibrio* | 16.90285 | -0.04661 | 2.66261 | -0.00194 | 1.00000 | 1.00000 |
| *Enterorhabdus* | 24.50976 | -0.08029 | 2.97524 | -0.00012 | 1.00000 | 1.00000 |
| *Slackia* | 31.56076 | 0.11102 | 2.98302 | 0.00000 | 1.00000 | 1.00000 |
| Supplement, treatment sequence, study phase age, and BMI included as fixed factors. | | | | | | |


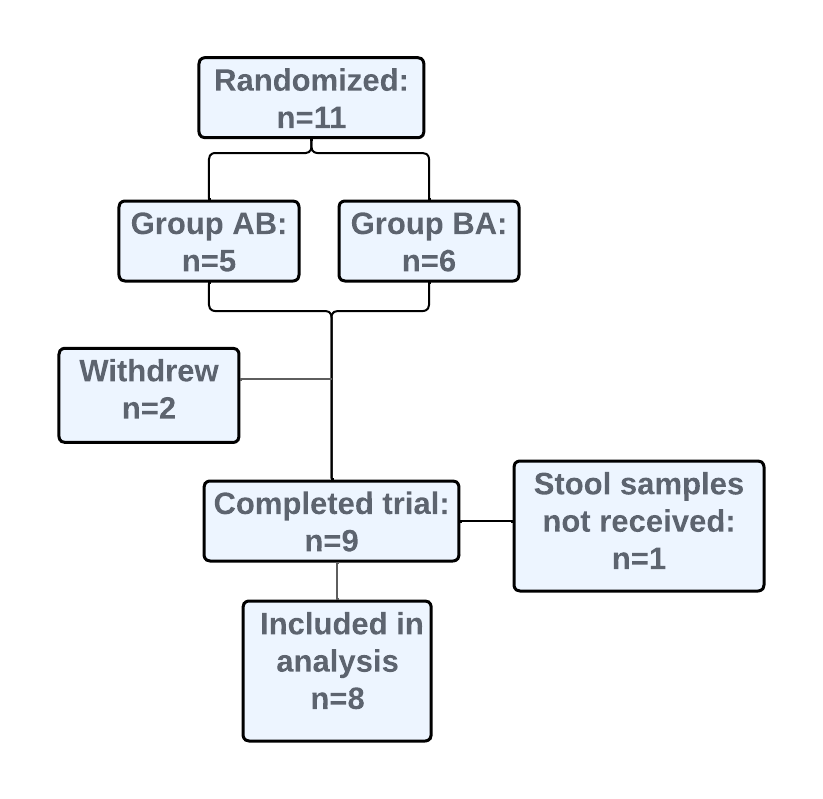


Supplemental Figure 1. Flow diagram of the progress through the randomized, double-blind, placebo-controlled, crossover study.
